# Editorial: Epidemiology, prevention, and control of animal diseases in Kazakhstan

**DOI:** 10.3389/fvets.2026.1783061

**Published:** 2026-01-29

**Authors:** Sarsenbay Abdrakhmanov, Andres M. Perez

**Affiliations:** 1 Institute of Animal Science and Veterinary, Seifullin Kazakh Agro Technical Research University, Astana, Kazakhstan; 2 Center for Animal Health and Food Safety, College of Veterinary Medicine, University of Minnesota, St Paul, MN, United States

**Keywords:** disease control, epidemiology, Kazakhstan, surveillance, veterinary sciences

With an area of 2,724,900 km^2^, the Republic of Kazakhstan is the ninth-largest country and the largest landlocked autonomous territory in the world. Located at multiple crossroads in Central Asia, Kazakhstan has historically been inhabited by nomadic communities. However, the country has experienced unprecedented political and economic changes over the last three centuries. Kazakhstan was incorporated into the Soviet Union through a long process that started with the gradual colonization by the Russian Empire in the 18th and 19th centuries and concluded with the annexation of its territory into the Soviet Union under the designation of the Kazakh Soviet Socialist Republic (KSSR) in 1936. The territory of the KSSR did not follow the traditional boundaries of the lands inhabited by the Kazakh people. For this reason, and due to other historical factors, the country that emerged after the declaration of independence on 16 December 1991 is home to a heterogeneous population. This heterogeneity, along with the country's complex history and the diversity of its vast territory's ecological, environmental, and demographic characteristics, has resulted in a variety of challenges and opportunities. Kazakhstan's modern sovereign statehood has been marked by an intention to advance the country by adjusting educational and research practices to those common in the Western Hemisphere. This Research Topic includes a collection of 12 articles that offer an overview of the recent history and current status of veterinary science, together with the prevention and control of animal diseases in Kazakhstan.

Two studies assessed the high-level evolution, status, and impact of animal diseases and veterinary science in Kazakhstan. Specifically, one discussed the impact that the Virgin Lands Campaign, implemented in the KSR between 1955 and 1970, may have had on the occurrence of foot-and-mouth disease (FMD) and anthrax in the country. The study highlighted the importance of systematic approaches in evaluating the design and planning of interventions (Mukhanbetkaliyev et al.). The second article explored the current state of veterinary science research in Kazakhstan, discussing needs, opportunities, progress, and gaps on the pathway that the country has followed to promote open science and research between 2018 and 2023 (Yessembekova et al.).

One study focused on companion animals, aiming to characterize canine parvovirus type 2, bacterial coinfections, and antimicrobial resistance profiles in Northern Kazakhstan (Aleshina et al.). Another study examined multiple species to assess the time-space dynamics of rabies between 2013 and 2023 and to identify the key drivers of transmission (Gomez-Buendia et al.).

The remaining eight studies were performed on production animals. Two contributions presented the results of cattle research, specifically aimed at evaluating and describing the epidemiological patterns of blackleg (Mussoyev et al.) and intestinal protozoa infections (Ussenbayev et al.). Another study presented the results of a serological surveillance study of trypanosomiasis, a disease found to be endemic in Kazakhstani camels (Abay et al.). One study provided an overview of epidemiological investigations of poultry infections in the country (Zikibayeva et al.). Finally, four manuscripts focused on sheep, including a review of the prevalence, genetic resistance, and associated factors of sheep diseases in Kazakhstan (Orkara et al.), epidemiological assessments, including phylogenetic analyses of Salmonella abortus ovis (Mussayeva et al.), sheep pox virus infections (Azanbekova et al.), and coenurosis (Kozhayeva et al.).

In summary, the 12 articles published in this Research Topic offer an overview of the pathway followed by Kazakhstan to promote open science, including an assessment of the historical impact of animal diseases in the country, the current status of veterinary science research, and the results of epidemiological studies conducted on companion and production animals. The contributions highlight the progress made by the country, stressing the need for continued investment in research and education in the region.

